# High-throughput quantification of osteogenesis in WJ-MSCs using Alizarin Red S, von Kossa, and Picro-Sirius red

**DOI:** 10.1080/20565623.2025.2567169

**Published:** 2025-10-01

**Authors:** Chung-You Huang, Win-Li Lin

**Affiliations:** Department of Biomedical Engineering, National Taiwan University, Taipei, Taiwan

**Keywords:** Alizarin red S, microplate reader, osteogenic differentiation, optical density values, Picro-Sirius red, von Kossa staining, Wharton’s Jelly mesenchymal stem cells

## Abstract

**Background:**

Osteogenic differentiation refers to the process by which mesenchymal stem cells (MSCs) transform into bone tissues. The microplate reader, characterized by its requirement for fewer samples, simple operation, excellent repeatability, and rapid detection speed, has been utilized to measure the absorbance of cells undergoing osteogenic differentiation.

**Research design and methods:**

In this study, we employed the microplate reader to analyze the histological stains on Wharton’s Jelly Mesenchymal Stem Cells (WJMSCs) in vitro, aiming to streamline future osteogenic differentiation studies.

**Results:**

It was not recommended to use a microplate reader to read Optical Density (OD) values for Alizarin red S and Picro-Sirius red (PSR) staining. However, a microplate reader could be effectively used to read OD values at wavelengths of 596, 620, and 680 nm from day 3 to day 5 in von Kossa staining (VKS) results.

**Conclusions:**

Thus, while the microplate reader is recommended for early-stage VKS quantification, its use is not advised for Alizarin red S staining (ARS) or PSR assays.

## PLAIN LANGUAGE SUMMARY

In this study, we looked for a faster and easier way to watch stem cells from the umbilical cord (from a part called Wharton’s Jelly) change into bone cells. This change is a process scientists call “osteogenic differentiation.”

To do this, we used a common lab machine called a microplate reader. This machine is great because it’s efficient and only needs tiny samples to work. We used it to analyze different colored dyes (stains) that scientists use to see when bone is starting to form.

Here’s what we discovered:

- The machine did not work well for measuring two specific stains: Alizarin red S and Picro-Sirius red.
- However, it worked very well for a different stain called von Kossa. The best time to take measurements was between days 3 and 5 of the process, using specific light settings (596, 620, and 680 nm).

In short, we found a quick and reliable method using a microplate reader and von Kossa staining. This new approach will help make future research on growing bone from these umbilical cord stem cells faster and more consistent.

## HIGHLIGHTS

Osteogenic differentiation is the key process whereby mesenchymal stem cells (MSCs) develop into bone tissue.

This study evaluates the use of a microplate reader for quantitative analysis of osteogenic differentiation in Wharton’s Jelly MSCs (WJMSCs) using histological stains.

The microplate reader is not recommended for quantifying Alizarin Red S (ARS) or Picro-Sirius Red (PSR) staining due to inconsistent results.

Effective quantification was achieved with von Kossa (VKS) staining, specifically at wavelengths of 596 nm, 620 nm, and 680 nm between days 3 and 5.

The findings support the use of microplate readers for early-stage, high-throughput assessment of mineralization via VKS staining in WJMSCs.

## 1. Introduction

A microplate reader, a laboratory instrument, is utilized to detect and measure biological, chemical, or physical events in microtiter plates, which contain multiple “wells” that function as small test tubes. This instrument can measure signals such as fluorescence, absorbance, luminescence, and time-resolved fluorescence. It finds extensive applications in enzyme-linked immunosorbent assays (ELISA), protein quantification, cell viability assays, among others. The microplate reader provides several benefits, such as the ability to process multiple samples concurrently, which saves time and reduces the consumption of samples and reagents. It also offers simple operation, excellent repeatability, quick detection speed, and high efficiency. However, it exhibits certain limitations, such as poor ultraviolet reading (wavelength < 300 nm), and necessitates the experimenter to possess advanced operational skills to ensure equal sample addition to each well of the microplate.

Osteogenic differentiation is the process that mesenchymal stem cells (MSCs) develop into osteoblasts to form and grow bone tissues [1]. This differentiation is essential for bone development, growth, repair, and regeneration [1]. The osteoblasts produce extracellular matrix proteins and paracrine factors to produce the organic constituents of the bone extracellular matrixes that facilitate their mineralization by inorganic compounds.

The microplate reader can be employed to measure the absorbance of cells that have undergone osteogenic differentiation [1,2].

Wharton’s Jelly MSCs (WJMSCs), a type of MSCs derived from the Wharton’s jelly in the umbilical cord [3], have potential applications in treating orthopedic injuries, promoting wound healing [4], curing spinal cord injuries and spinal stenosis, and improving functional recovery after traumatic brain injury.

WJMSCs could differentiate into osteogenic, adipo-genic, chondrogenic, and other lineages [5]. Stimulation of the bone morphogenetic protein 2 (BMP-2) pathway enhances the osteogenic differentiation of WJMSCs. This method, like the differentiation process of Bone Marrow MSCs (BM-MSCs), positions WJMSCs as a valuable alternative to BM-MSCs [6].

WJMSCs have demonstrated significant osteogenic potential in vitro. Under appropriate induction conditions—typically containing dexamethasone, ascorbic acid, and β-glycerophosphate—WJMSCs differentiate into osteoblast-like cells, expressing osteogenic markers and forming mineralized matrix, as evidenced by Alizarin Red S (ARS) staining [7–9]. These properties highlight the promise of WJMSCs in bone tissue engineering and regenerative medicine [7–9].

The von Kossa staining (VKS), ARS staining, and Picro-Sirius red staining (PSR staining) are all histological staining methods on tissue sections. VKS is employed to visualize mineralization in tissues, such as calcium and potassium [10]. This staining method involves treating tissue sections with a silver nitrate solution to replace calcium with silver deposits, which can be visualized as metallic silver. ARS staining chelates calcium ions to form orange-red complexes, enabling visualization and quantification of calcium-rich nodules [11–13]. PSR specifically stains collagen fibers and, under polarized light, enhances birefringence for detailed assessment of collagen deposition—a key indicator of extracellular matrix maturation during osteogenic and chondrogenic differentiation [14].

The microplate reader, known for its ability to process multiple samples simultaneously and its rapid detection speed, along with WJMSCs, recognized for their potential as a valuable alternative to Bone Marrow MSCs (BM-MSCs) in osteogenic differentiation [6], can be effectively employed in osteogenic differentiation studies. Additionally, histological staining methods, due to their cost-effectiveness and capability to handle large samples, present significant advantages.

In this study, we utilized a microplate reader to analyze the histological stains on WJMSCs in vitro thereby facilitating future studies on osteogenic differentiation.

In this study, we employed a microplate reader to quantitatively analyze VKS, ARS, and PSR staining in WJMSC cultures, providing a reliable and high-throughput method to facilitate future research on osteogenic differentiation.

## 2. Material and methods

### 2.1. Cells

WJMSCs were obtained from BCRC (No. RM60596; Hsinchu, Taiwan). The cells were cultured Dulbecco’s Modified Eagle’s Medium low glucose (DMEM low glucose; HyClone^™^, United States) supplemented with 10% fetal bovine serum (FBS, Gibco^™^, United States), and 1% antibiotic antimycotic (Pen/Strep/Fungizone) solution (AS, HyClone^™^, United States) at 37 °C in an incubator with 5% CO_2_. The medium was changed every 2–3 days. For ARS, VKS, and PSR staining, approximately 320,000 cells were seeded per well in 6-well plates and cultured under basic medium conditions without induction agents or medium replacement throughout the experiment.

### 2.2. Staining and sample processing

At the end of the culture period, the medium was aspirated and the cells were gently rinsed twice with pre-warmed phosphate-buffered saline (PBS). The cells were then fixed with 4% paraformaldehyde (PFA) in PBS for 15 minutes at room temperature. After fixation, the PFA solution was removed and the fixed cells were washed three times with PBS (5 minutes per wash) to remove residual fixative.

This study employed Alizarin Red S (ARS), von Kossa (VKS), and Picro-Sirius Red (PSR) staining to assess osteogenic differentiation. ARS and VKS were used to detect calcium deposition, while PSR was applied to visualize collagen fibers. No additional pretreatment was necessary for VKS or PSR staining.

For ARS staining, which targets calcium mineralization, fixed samples were rinsed with deionized water after PBS washes to eliminate phosphate ions that could interfere with staining, and then air-dried completely. The ARS staining solution (2%, pH 4.2) was prepared using alizarin red S sodium salt (Alfa Aesar^®^, UK), which appears as an orange crystalline powder. The fixed cells were then stained with this solution for 10 minutes at room temperature to detect calcium mineralization. After staining, the samples were thoroughly rinsed with deionized water to remove unbound dye and air-dried completely before imaging and quantification.

The Von Kossa staining was performed using a commercial kit (BioTnA, Taiwan) visualize calcium deposits (gray), nuclei (red), and cytoplasm (light pink). Fixed cells were treated with 5% silver nitrate solution under UV light for 30 minutes at room temperature. Subsequently, the samples were rinsed with deionized water, treated with 5% sodium thiosulfate for 5 minutes to remove unreacted silver, and washed again with deionized water before drying.

The Picro Sirius Red Stain Kit (Abcam, UK) was used according to the manufacturer’s instructions to identify collagen deposition. Briefly, fixed cells were stained with 0.1% Sirius Red in saturated picric acid for 1 hour at room temperature. This method enables the visualization of total collagen (red), muscle fibers (yellow), cytoplasm (yellow). When examined under polarized light, type I collagen exhibits yellow-orange birefringence, while type III collagen displays green birefringence. After staining, the samples were rinsed twice with acidified water (0.5% acetic acid) to remove unbound dye, followed by dehydration through a graded ethanol series prior to imaging.

Staining results were quantified using a BioTek Synergy HTX microplate reader. The peak absorbance wavelengths for ARS were determined to be 556 nm and 596 nm. For VKS staining, readings were taken at 620 nm for nuclei (red) and 680 nm for calcium deposits (gray). In PSR staining, wavelengths of 556 nm, 596 nm, and 680 nm were selected to detect nuclei, type III collagen (green birefringence), type I collagen (yellow-orange birefringence), and total collagen (red), respectively.

### 2.3. Image capture

Images were captured using an inverted light microscope equipped with a digital camera. Brightfield images were taken at 4× magnification from the objective lens. Due to the absence of a blue light filter, color balance was adjusted during image processing using ImageJ software by modifying the blue channel threshold (minimum value increased from 0 to 128) to reduce brightness and improve contrast.

### 2.4. Analysis

Data analysis and graphical representation were conducted using MATLAB R2016b (MathWorks, Natick, MA, USA). Statistical analyses included the calculation of mean values, standard deviations, and coefficients of variation (CV) to assess data variability and reliability. Significant differences between time points (e.g., Day 3 vs. Day 5, Day 3 vs. Day 10) were evaluated using appropriate statistical tests, such as t-tests or ANOVA, based on data distribution and experimental design.

Additionally, ImageJ software (National Institutes of Health, Bethesda, MD, USA) was employed for image processing and quantitative analysis of staining results. This included adjusting color balance and brightness, particularly for images captured without a blue light filter, by modifying color scale thresholds (e.g., increasing the minimum blue value from 0 to 128) to improve visualization and accuracy.

## 3. Results and discussion

**Figure 1.**
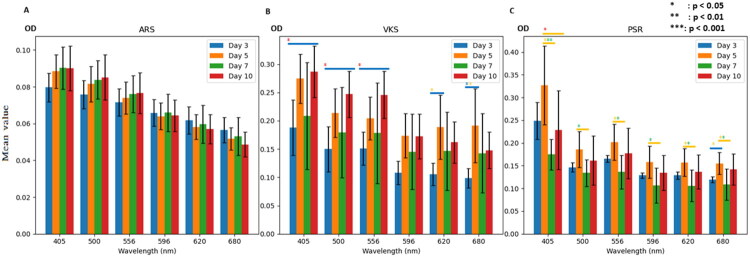
The mean OD values of different wavelengths (405, 500, 556 596, 620 & 680 nm) from day 3 to 10 in different stains with error bar. (A) Alizarin Red S staining. (B) Von Kossa staining. (C) Picro-Sirius red staining. OD: optical density.

Figure 1 presented the mean results from Day 3 to Day 10. In the ARS staining results, the mean optical density (OD) values at 500 nm and 556 nm increased with the progression of days. Similarly, the mean OD value at 405 nm also increased until Day 7. The VKS indicated that the mean OD values at 405 nm, 500 nm, and 556 nm exhibited a trend of increasing with the number of days. Additionally, the mean OD values at 596 nm, 620 nm, and 680 nm also increased until Day 5. The PSR staining results displayed a pattern resembling a counterclockwise 45° “Z” across all wavelengths.

**Figure 2.**
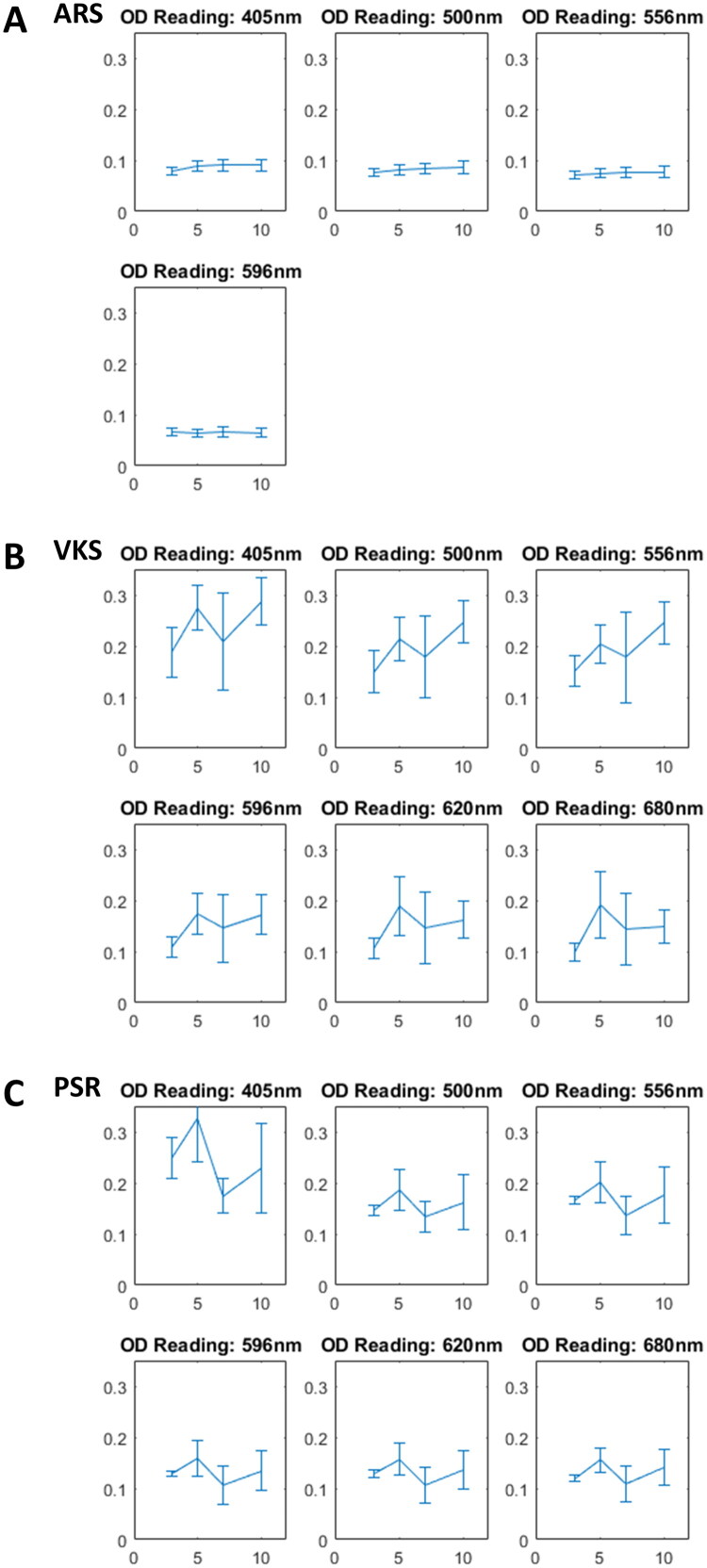
The OD results with SD from day 3 to 10. (A) Alizarin Red S staining (ARS). (B) Von Kossa staining (VKS). (C) Picro-Sirius red staining (PSR). OD: optical density; SD: standard deviation.

Incorporating the standard deviation into the analysis (Figure 2), no significant differences were observed in the OD values from Day 3 to Day 10 at wavelengths of 405 nm, 500 nm, 556 nm, and 596 nm in the ARS staining results. In the VKS results, two significant differences were identified. The first significant difference was observed between Day 3 and Day 10 at wavelengths of 405 nm, 500 nm, 556 nm, 596 nm, and 680 nm. The second significant difference was noted between Day 3 and Day 5 at wavelengths of 596 nm, 620 nm, and 680 nm.

Additionally, a significant difference in OD values was found between Day 3 and Day 10 at a wavelength of 680 nm in the PSR staining results. Upon further consideration of the coefficient of variation (CV) (Tables 1 and 2), a good CV was observed between Day 3 and Day 10 at wavelengths of 405 nm, 500 nm, 556 nm, 596 nm, and 680 nm in the VKS results. A good CV was also found between Day 3 and Day 5 at wavelengths of 596 nm and 620 nm in the VKS results. In addition to the VKS results, a good CV was observed between Day 3 and Day 10 at a wavelength of 680 nm in the PSR staining results.

**Table 1.** The summary results of ARS, VKS, and PSR.

The summary results of ARS, VKS, and PSR.												
OD read	DAY3			DAY5			DAY7			DAY10		
	M	SD	CV	M	SD	CV	M	SD	CV	M	SD	CV
ARS	P13, *n* = 6			P13-14, *n* = 12			P13-14, *n* = 18			P13-14, *n* = 12		
405 nm	0.0797	0.0078	9.74%	0.0884	0.0091	10.33%	0.0902	0.0115	12.70%	0.0901	0.0122	13.58%
500 nm	0.0758	0.0076	10.02%	0.0815	0.0095	11.72%	0.0838	0.0105	12.49%	0.0852	0.0123	14.48%
556 nm	0.0717	0.0074	10.35%	0.0739	0.0087	11.73%	0.0760	0.0101	13.27%	0.0767	0.0111	14.43%
596 nm	0.0658	0.0072	10.97%	0.0639	0.0073	11.37%	0.0659	0.0102	15.52%	0.0643	0.0086	13.38%
620 nm	0.0618	0.0074	11.90%	0.0582	0.0067	11.54%	0.0596	0.0103	17.31%	0.0570	0.0078	13.75%
680 nm	0.0565	0.0069	12.25%	0.0518	0.0061	11.72%	0.0531	0.0104	19.61%	0.0487	0.0067	13.83%
VKS	P13, n = 6			P14, n = 6			P13-14, n = 18			P13-14, n = 12		
405 nm	0.1880	0.0488	25.93%	0.2745	0.0434	15.80%	0.2085	0.0944	45.30%	0.2867	0.0459	16.00%
500 nm	0.1498	0.0402	26.84%	0.2137	0.0427	19.96%	0.1791	0.0799	44.60%	0.2469	0.0408	16.52%
556 nm	0.1508	0.0293	19.39%	0.2045	0.0377	18.46%	0.1783	0.0888	49.81%	0.2459	0.0415	16.89%
596 nm	0.1082	0.0205	18.92%	0.1735	0.0391	22.52%	0.1453	0.0667	45.89%	0.1724	0.0392	22.72%
620 nm	0.1053	0.0197	18.73%	0.1888	0.0565	29.93%	0.1464	0.0692	47.24%	0.1619	0.0363	22.43%
680 nm	0.0987	0.0172	17.47%	0.1913	0.0657	34.32%	0.1428	0.0698	48.92%	0.1478	0.0323	21.86%
PSR	P14, *n* = 6			P14, *n* = 12			P13-14, *n* = 18			P13-14, *n* = 12		
405 nm	0.2488	0.0405	16.29%	0.3272	0.0867	26.50%	0.1746	0.0336	19.27%	0.2285	0.0866	37.90%
500 nm	0.1467	0.0103	7.04%	0.1854	0.0397	21.39%	0.1340	0.0298	22.22%	0.1617	0.0536	33.17%
556 nm	0.1657	0.0078	4.69%	0.2017	0.0397	19.67%	0.1364	0.0368	26.96%	0.1772	0.0553	31.21%
596 nm	0.1287	0.0058	4.47%	0.1578	0.0350	22.19%	0.1064	0.0382	35.92%	0.1344	0.0388	28.84%
620 nm	0.1288	0.0080	6.24%	0.1573	0.0303	19.26%	0.1060	0.0347	32.72%	0.1366	0.0374	27.37%
680 nm	0.1192	0.0065	5.45%	0.1552	0.0242	15.61%	0.1090	0.0341	31.29%	0.1415	0.0341	24.12%

ARS: Alizarin red S; CV: coefficient of variation; M: mean; OD: optical density; P: passenger; PSR: Picro-Sirius red; SD: standard deviation; VKS: von Kossa stain.

**Table 2.** The results of ARS, VKS, and PSR with good CV (<30%) or application in the potential from Table 1.

The results of ARS, VKS, and PSR with good CV (<30%) or application in the potential												
OD read	DAY3			DAY5			DAY7			DAY10		
	M	SD	CV	M	SD	CV	M	SD	CV	M	SD	CV
ARS	P13, *n* = 6			P13-14, *n* = 12			P13-14, *n* = 18			P13-14, *n* = 12		
556 nm	0.0717	0.0074	10.35%	0.0739	0.0087	11.73%	0.0760	0.0101	13.27%	0.0767	0.0111	14.43%
596 nm	0.0658	0.0072	10.97%	0.0639	0.0073	11.37%	0.0659	0.0102	15.52%	0.0643	0.0086	13.38%
VKS	P13, n = 6			P14, n = 6			P13-14, n = 18			P13-14, n = 12		
405 nm	0.1880	0.0488	25.93%							0.2867	0.0459	16.00%
500 nm	0.1498	0.0402	26.84%							0.2469	0.0408	16.52%
556 nm	0.1508	0.0293	19.39%							0.2459	0.0415	16.89%
596 nm	0.1082	0.0205	18.92%	0.1735	0.0391	22.52%				0.1724	0.0392	22.72%
620 nm	0.1053	0.0197	18.73%	0.1888	0.0565	29.93%				0.1619	0.0363	22.43%
680 nm	0.0987	0.0172	17.47%							0.1478	0.0323	21.86%
PSR	P14, *n* = 6			P14, *n* = 12			P13-14, *n* = 18			P13-14, *n* = 12		
556 nm	0.1657	0.0078	4.69%	0.2017	0.0397	19.67%	0.1364	0.0368	26.96%			
596 nm	0.1287	0.0058	4.47%	0.1578	0.0350	22.19%				0.1344	0.0388	28.84%
680 nm	0.1192	0.0065	5.45%	0.1552	0.0242	15.61%				0.1415	0.0341	24.12%

ARS: Alizarin red S; CV: coefficient of variation; M: mean; nm: nanometer; OD: optical density; P: passenger; PSR: Picro-Sirius red; SD: standard deviation; VKS: von Kossa stain.

**Figure 3.**
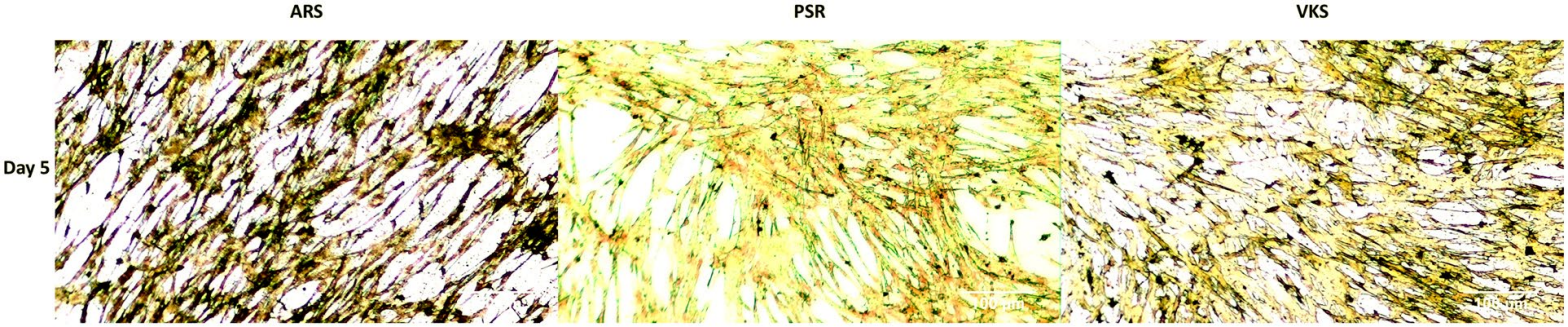
The image results for Alizarin red S (ARS) stain, von Kossa stain (VKS), and Picro-Sirius red (PSR) stain were obtained on days 5. The objective lens had a magnification of 4X. The scale bars represented 100 μm.

Figure 3 presented the image results of ARS, VKS, and PSR at Day 5. Due to the absence of a blue light filter in the utilized microscopy, the image was adjusted for color balance by increasing the minimum of the blue color scale from 0 to 128 to reduce brightness.

Based on Figure 1A, an absorbance wavelength of 556 nm was suitable for ARS to measure calcium deposits from day 3 to day 10. The OD values at 405 nm and 500 nm increased with the number of days until day 7 and day 10, respectively. In VKS results (Figure 1B), the absorbance wavelengths of 620 nm and 680 nm were suitable for reading nuclei (red) and deposited calcium (gray) from day 3 to day 5, respectively. The mean OD value at 596 nm also increased with the number of days until day 5. The means of OD values at 405, 500, and 556 nm showed a trend of increasing with the number of days. The PSR staining results resembled a counterclockwise 45-degree Z-shape across all wavelengths, which included 556, 596, and 680 nm for reading nuclei type III collagen (green birefringence), type I collagen (yellow-orange birefringence), and collagen (red), respectively.

Although there were good CV in OD values from day 3 to day 10 on the wavelengths of 556 and 596 nm in ARS staining results with less effect by the phenomenon of green color at day 5, the lack of significant differences makes it less recommended to use a microplate reader to read ARS results. The OD values at day 5 were higher than those at day 7 across all absorbance wavelengths in VKS and PSR results. Due to the lack of images at day 10, it was highly recommended to use a microplate reader to read OD values between day 3 and day 5 on the wavelengths of 596, 620, and 680 nm in VKS results.

The findings from this study demonstrate that the microplate reader can effectively quantify certain histological stains associated with osteogenic differentiation, though its utility varies depending on the staining method and wavelength. The increasing OD values in ARS and VKS staining over time suggest progressive mineralization, consistent with known osteogenic differentiation processes [1,7–9]. However, the lack of significant differences in ARS results across most wavelengths indicates limited sensitivity for detecting early mineralization events, which may be due to the low calcium deposition levels in the early stages of differentiation [11–13]. In contrast, VKS staining showed significant differences and good coefficients of variation at specific wavelengths (e.g., 596 nm, 620 nm, 680 nm) between Days 3 and 5, suggesting that the microplate reader is more suitable for quantifying phosphate deposits during early osteogenic differentiation [10]. The decrease in OD values after Day 5 in VKS and PSR may reflect saturation of staining or matrix remodeling, which warrants further investigation [14]. The Z-shaped pattern in PSR results may indicate complex collagen dynamics, potentially involving shifts in collagen type composition during differentiation [14]. These results underscore the importance of selecting appropriate staining methods and wavelengths when using a microplate reader for quantitative analysis of osteogenic differentiation. Future studies should include longer time points and correlate microplate reader data with imaging techniques to validate these findings (Tables 3 and 4).

**Table 3.** Summary of ANOVA results.

Staining method	OD read (nm)	*F* value	*p* value	Significant (*p* < 0.05)	Total sample size	df (between)	df (within)
PSR	405	9.80354	0.000064	Yes	42	3	38
	500	3.60605	0.021879	Yes	42	3	38
	556	5.03134	0.004924	Yes	42	3	38
	596	4.38036	0.009617	Yes	42	3	38
	620	5.22723	0.004041	Yes	42	3	38
	680	5.81891	0.002249	Yes	42	3	38
VKS	405	4.33211	0.010114	Yes	42	3	38
	500	4.44055	0.009033	Yes	42	3	38
	556	3.7085	0.019593	Yes	42	3	38
	596	2.49119	0.074816	No	42	3	38
	620	2.55215	0.069867	No	42	3	38
	680	2.79749	0.05311	No	42	3	38
ARS	405	1.58685	0.206074	No	48	3	44
	500	1.20147	0.320358	No	48	3	44
	556	0.46196	0.710269	No	48	3	44
	596	0.16146	0.921706	No	48	3	44
	620	0.49221	0.689511	No	48	3	44
	680	1.35076	0.270267	No	48	3	44

ARS: Alizarin red S; df: degree of freedom; nm: nanometer; PSR: Picro-Sirius red; OD: optical density; p: p-value; VKS: von Kossa stain.

**Table 4.** Summary of significant Tukey HSD Post-Hoc test results (only significant comparisons are listed, *p* < 0.05).

Staining method	OD read (nm)	Significant comparison	*Q* value	*p* value
PSR	405	DAY5:DAY7	6.76	0.00015
		DAY5:DAY10	4.37	0.01865
	500	DAY5:DAY7	4.03	0.03376
	556	DAY5:DAY7	4.85	0.00768
	596	DAY5:DAY7	4.56	0.01314
	620	DAY5:DAY7	4.96	0.00623
	680	DAY3:DAY5	3.83	0.04772
		DAY5:DAY7	4.91	0.00682
VKS	405	DAY3:DAY10	4.00	0.03586
	500	DAY3:DAY10	4.59	0.01251
	556	DAY3:DAY10	4.21	0.02502
	620	DAY3:DAY5	4.44	0.01645
	680	DAY3:DAY5	4.85	0.00772

Nm: nanometer; PSR: Picro-Sirius red; OD: optical density; p: *p* value; VKS: von Kossa stain.

## 4. Conclusions

Although the microplate reader offers benefits such as ease of use, high reproducibility, rapid detection, and efficiency, its application for quantifying ARS staining via OD measurement was not advised due to minimal intergroup differences. In both VKS and PSR assays, OD values measured at all applicable wavelengths were higher on day 5 compared to day 7. It is proposed that the microplate reader could be suitably employed for OD measurement in VKS staining between days 3 and 5 at wavelengths of 596 nm, 620 nm, and 680 nm—where 620 nm and 680 nm correspond to nuclei and calcium deposits, respectively. Conversely, the use of the microplate reader for OD quantification in PSR staining is not recommended.

### 4.1. Future perspective

Building on these findings, future research should prioritize the development of wavelength-optimized detection protocols to enhance the specificity of microplate readers in monitoring early osteogenic differentiation (days 3–5) in von Kossa staining. Translating these wavelength- and time-dependent thresholds into standardized 3D bone models or patient-specific MSC platforms will accelerate clinical translation, enabling personalized screening for bone regenerative therapies.

## Data Availability

The data that support the findings of this study are available from the corresponding author upon reasonable request.

## Writing assistance

No writing assistance was utilized in the production of this manuscript
